# Improving animal research reporting standards: HARRP, the first step of a unified approach by ICLAS to improve animal research reporting standards worldwide

**DOI:** 10.15252/embr.201846069

**Published:** 2018-04-18

**Authors:** Nikki Osborne, Marc T Avey, Lida Anestidou, Merel Ritskes‐Hoitinga, Gilly Griffin

**Affiliations:** ^1^ Royal Society for the Prevention of Cruelty to Animals Southwater UK; ^2^ Ottawa Hospital Research Institute Ottawa ON Canada; ^3^ Institute for Laboratory Animal Research National Academies of Sciences, Engineering and Medicine Washington DC USA; ^4^ SYstematic Review Center for Laboratory animal Experimentation (SYRCLE) Radboud University Medical Center Nijmegen The Netherlands; ^5^ Canadian Council for Animal Care Ottawa ON Canada

**Keywords:** Methods & Resources, S&S: Ethics, S&S: Politics, Policy & Law

## Abstract

The HAARP guidelines aim to set a global minimum standard for reporting results from and details of research experiments using animals. Their adoption would contribute to more transparency in research and improve reproducibility.

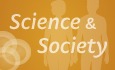

For more than 30 years, individuals and organizations have expressed concerns about the quality of reporting the results from and details of research experiments that use animals. These concerns and efforts to establish better standards along with guidelines for researchers (see Sidebar A) have gained more attention and importance lately given the ongoing discussion about a “reproducibility crisis” in biomedical research along with similar efforts to further improve the welfare of laboratory animals. However, implementation of reporting standards by journals and adherence to these by authors is still patchy. Given this variation in awareness and implementation of current reporting standards, the International Council for Laboratory Animal Science (ICLAS) decided to seek to harmonize animal research reporting guidelines so as to encourage improvements in the quality of science where laboratory animals are involved. ICLAS, which was formed under the auspices of UNESCO in 1956 as an international scientific organization to advance human and animal health by promoting the ethical care and use of laboratory animals in research worldwide, believes that improving research reporting will aid the dissemination of responsible research practices worldwide and reduce the impact of cultural factors influencing the ethical use of animals. Here, we present simplified and general reporting principles that would make it easier for both journals and authors to report details of animal experiments. Adoption and implementation of these general principles could improve reproducibility of research results and animal welfare globally.

**Sidebar A: Further reading**

**Papers outlining concerns regarding the reporting of animal experiments**

Smith JA, Birke L, Sadler D (1997) Reporting animal use in scientific papers. *Lab Animal* 31: 312–317

Alfaro V (2005) Specification of laboratory animal use in scientific articles: current low detail in the journals’ instructions for authors and some proposals. *Methods Find Exp Clin Pharmacol* 27(7): 495–502

Phillips CJC (2005) Meta‐analysis – a systematic and quantitative review of animal experiments to maximise the information derived. *Anim Welf* 14(4): 333–338

Mignini LE, Khan KS (2006) Methodological quality of systematic reviews of animal studies: a survey of reviews of basic research. *BMC Med Res Methodol* 6: 10

Kilkenny C, Parsons N, Kadyszewski E, Festing MFW, Cuthill IC, Fry D *et al* (2009) Survey of the quality of experimental design, statistical analysis and reporting of research using animals. *PLoS One* 4: e7824

Landis SC, Amara SG, Asadullah K, Austin CP, Blumenstein R, Bradley EW *et al* (2012) A call for transparent reporting to optimize the predictive value of preclinical research. *Nature* 490: 187–191

Osborne NJ, Payne D, Newman ML (2009) Journal editorial policies, animal welfare, and the 3Rs. *Am J Bioeth* 9: 55–59

Taylor K (2010) Reporting the implementation of the Three Rs in European primate and mouse research papers: Are we making progress? *Altern Lab Anim* 2010; 38: 495–517

Muhlhausler BS, Bloomfield FH, Gillman MW (2013) Whole Animal Experiments Should Be More Like Human Randomized Controlled Trials. *PLoS Biol* 11: e1001481

**Papers providing guidance on the reporting of experiments involving animal use**

Ellery AW (1985) Guidelines for specification of animals and husbandry methods when reporting the results of animal experiments. Working Committee for the Biological Characterization of Laboratory Animals/GV‐SOLAS. *Lab Anim* 19(2): 106–108

Festing MFW, van Zutphen LFM (1997) Guidelines for reviewing manuscripts on studies involving live animals: Synopsis of the workshop. In *Animal Alternatives, Welfare and Ethics*, van Zutphen LFM, Balls M (eds), pp 405–410. New York: Elsevier

Brattelid T, Smith AJ (2000) Guidelines for reporting the results of experiments on fish. *Lab Anim* 34: 131–135

Festing MFW, Altman DG (2002) Guidelines for the design and statistical analysis of experiments using laboratory animals. *ILAR J* 43: 244–258

Taylor CF, Field D, Sansone S, Aerts J, Apweiler R *et al*. (2008) Promoting coherent minimum reporting guidelines for biological and biomedical investigations: the MIBBI project. *Nat Biotechnol* 26: 889–896

Hooijmans CR, Leenaars M, Ritskes‐Hoitinga M (2010) A gold standard publication checklist to improve the quality of animal studies, to fully integrate the three Rs, and to make systematic reviews more feasible. *Altern Lab Anim* 38: 167–182

Kilkenny C, Browne WJ, Cuthill IC, Emerson M, Altman DG. (2013) Improving bioscience research reporting: The arrive guidelines for reporting animal research. *Animals* 4: 35–44

National Research Council (2011) *Guidance for the description of animal research in scientific publications*. Washington, DC: National Academies Press

National Institutes of Health (2015) Principles and Guidelines for Reporting Preclinical Research. http://www.nih.gov/about/reporting-preclinical-research.htm

Editorial (2013) Announcement: Reducing our irreproducibility. *Nature* 496: 398–398

Pulverer B (2014) Transparent, reproducible data. *EMBO J* 33: 2597

## Reporting guidelines for research with animals

The first specific guidance on reporting “animals and husbandry methods” was published in 1985 (see Sidebar A for an overview on animal reporting guidelines). In 1997, Michael Festing and colleagues published a checklist based on a workshop of journal editors discussing how to review manuscripts on studies involving live animals. More recently, the Institute for Laboratory Animal Research's (ILAR) *Guidance for the description of animal research in scientific publications* describes what should be reported in the Methods section of a research paper. Festing and Altman's guidelines adopt an alternative approach that emphasizes the importance of experimental design and statistical analysis in promoting efficient and humane animal‐based research, including addressing ethical concerns; minimizing waste, particularly of animal lives; and enabling extraction and subsequent reporting of useful information. Alfaro also focused on the practice of scientifically and ethically acceptable research, but for the first time consolidated the different pieces into the standard IMRAD (Introduction, Methods, Results and Discussion) structure of manuscripts to improve implementation. By 2008, a number of collaborative efforts focused on minimum information reporting guidelines to improve the quality of research articles over a wide range of experimental designs and analytical techniques, with and without animal experimentation. To bring these efforts together, the Minimum Information for Biological and Biomedical Investigations (MIBBI) project was launched to collate all the reporting guidelines and devise a standard protocol for their development so that they are compatible with each other (http://www.fairsharing.org).

… the HARRP could be used as an easily translatable minimum standard, upon which a more structured framework could be built.

In 2009, Nikki Osborne and colleagues reported that only 53% of journals publishing research involving animal experiments had an editorial policy or guidelines on reporting (Sidebar A). Most of these policies added little or no value in terms of the quality of information reported: many just included the word “animal” or requested that research conforms to legal standards. Thus, in the 20 or more years since the first guidelines were published, implementation was still lagging. A survey by Kilkenny and colleagues looking at how experimental design and statistical analysis were reported in published biomedical research involving animal use, identified a number of quality and reporting issues—that had been discussed since 1997—reinforcing the fact that nothing had changed.

Carlijn Hooijmans and colleagues identified the poor quality of animal research reporting as a critical factor impeding clinical researchers from systematically reviewing preclinical animal research‐derived data (Sidebar A). To improve the translatability of preclinical or basic studies as a basis for clinical trials, the “Gold Standard Publication Checklist” was published to make systematic reviews and meta‐analysis of animal studies more practical while promoting greater implementation of the 3Rs (reduce, refine and replace) principles of humane experimental technique 1. This was quickly followed by the “Animals in Research: Reporting In Vivo Experiments” (ARRIVE) guidelines, the focus of which was to maximize the usefulness of published research. The ARRIVE guidelines, the result of the work of an expert group of scientists, statisticians, journal editors and research funders, are based upon the CONSORT statement for reporting randomized controlled clinical trials 2. Again, it was thought that a standard reporting format would help to facilitate their implementation; however, despite being endorsed by more than 1,000 journals, their implementation and enforcement remain challenging 3 and a review is currently underway to determine the impact ARRIVE has had on animal research reporting to date (https://ecrf1.clinicaltrials.ed.ac.uk/iicarus).

## The ICLAS working group on harmonization of reporting guidelines

Given the great variation in the level of awareness and implementation by the research community and journals of the three main animal research reporting standards (the Gold Standard Publication Checklist, the ARRIVE guidelines and ILAR's Guidance on the Description of Animal Research in Scientific Publications), ICLAS attempted to further harmonize animal research reporting guidelines. Representatives of the groups with guidelines and additional experts in research reporting were invited to participate in a “Working Group to Harmonize the Reporting of Animal Research”, charged with identifying key principles consistent across each guidance document for developers of health research reporting guidelines 4 and data synthesis methods 5.

Soon after the working group commenced, the participants of a meeting held at the US National Institute of Neurological Disorders and Stroke (NINDS) in June 2012 called for “transparent reporting to optimize the predictive value of preclinical research” and proposed “a core set of reporting standards for rigorous study design”. Shortly thereafter, the Nature Publishing group announced the introduction of their own checklist to improve the reproducibility and reporting standards of research published in their journals. Similarly, EMBO Press published its own checklist to standardize the reporting of key information so as to support re‐analysis and repetition of experiments by the scientific community. These are consistent with another NIH initiative based on a 2014 meeting between funders and journals resulting in the “Principles and Guidelines for Reporting Preclinical Research” (Sidebar A).

## The harmonized animal research reporting principles

The work of the ICLAS working group has resulted in eight harmonized animal research reporting principles (HARRP; see Boxes 1 and 2), based on a comparative analysis of the ARRIVE guidelines, the GSPC and the ILAR Guidance, with further development following a process similar to that of the CONSORT standards for reporting clinical trials 2, 4, 5. Six of the key principles were agreed upon: ethics; background and objectives; study design; animal details; experimental protocol; and details of housing/husbandry and research environment. These principles along with a comment form and background letter were then submitted to 11 journal editors for comments. Based on the feedback from 13 editors (some of the editors contacted originally passed the email along to additional colleagues), the reporting principles were updated to include: “conflict of interests” and “data availability”.

**Box 1: Animal research reporting principles**

- Ethics: Confirmation that an ethical review was conducted prior to the research being conducted must be mandatory in all publications where animals are used in research.
- Funding and conflict of interests: Funding sources for animal‐based research and conflict of interest for any of the authors named in the publication must be reported.
- Background and scientific objectives: For animal research, there must be sufficient scientific background to explain the rationale for the experimental approach, and a clear explanation of how and why the particular animal species and model are the most appropriate to address the scientific objectives.
- Study design: All published animal studies must include sufficient detail to facilitate critical review of the methods and results presented.
- Animal subjects: The source and details of animal subjects in research as well as the experimental characteristics that are monitored and recorded for the purposes of the study must be included in publications.
- Experimental protocols: Details of experimental protocols must be reported and should include details of any procedures and materials related to the humane treatment and welfare of the animals.
- Housing, husbandry and research environment: Housing, husbandry and all other non‐experimental research environmental factors related to animal‐based research must be reported.
- Data availability: All animal‐derived *in vitro* or *ex vivo* data must be made available.

**Box 2: HARRP in practice**

- Ethics: The ethical statement should include four parts:
  a clear statement indicating that all animal use in the study received prior approval;
  the name and location of the ethics review board(s) that approved the study;
  all national, local and/or international regulations and guidelines that the study has complied with or has specific exemption from; and
  all license, permit and protocol identifiers associated with the approvals.
- Funding and conflict of interests: The conflict of interest statement should include four parts:
  the name of all sources of funding and other support;
  the identifiers for all funding/support sources (e.g. grant code or equivalent);
  the role of all funders/support in the study; and
  a conflict of interest statement for all authors.
- Background and scientific objectives: The background should include four parts:
  a description of the scientific background and rationale;
  a description and justification of both the animal species and model;
  an explanation of the expected findings’ generalizability or translation; and
  the scientific hypotheses/objectives and all outcomes (primary and secondary).
- Study design: The study design should include the following:
  the total number of animals and how the sample size was estimated (e.g. sample size calculation);
  the number of experimental and control groups for all experiments including the total (absolute) number of animals in each of these groups;
  the methods used to reduce bias when assigning animals to groups (randomization, allocation concealment and/or others) as well as how the personnel were blinded during the conduct of the study and assessment of results;
  a description of the experimental unit and any inclusion/exclusion criteria; and
  a description of each statistical test used including the unit of analysis and an explanation for why any data were excluded.
- Animal subjects: The description of the animals should include the following:
  the species;
  strain;
  sex;
  age (mean/median + range);
  weight (mean/median + range);
  international genetic nomenclature;
  the source of the animals;
  health status; and
  the baseline age (mean/median + range) and weight (mean/median + range) for all groups.
- Experimental protocols: The experimental procedures should describe the following:
  the drug/vehicle formulation, doses, site, route of administration, the frequency including the time between and order of doses as well as the time between dose and sampling;
  all use of anesthesia and analgesia including doses, site and route of administration;
  all surgical procedures, including equipment and all monitoring procedures;
  the method of euthanasia including, if applicable, the dose, site and route of administration;
  all materials and equipment used including vendor and catalogue numbers or equivalent; and
  adverse events during all stages of the experiment, for all groups.
- Housing, husbandry and research environment: Housing, husbandry and research environment should include the following:
  the type of facility;
  the type of housing and cage including the bedding material;
  the number of animals per cage:
  light/dark cycle;
  temperature;
  type of food and access to food; and
  any environmental enrichment. These items should be reported for housing prior to the experiment and anytime that animals are returned to housing during the experiment (e.g. after surgery but prior to treatment).
- Data availability: Data availability should require that
  all data be available (when legally and ethically appropriate) for review and analysis either during or after the publication; and
  data be deposited into a public repository and linked (e.g. identification number in database and doi of published manuscript) to ensure data can be located.

The eight principles are listed in Box 1, while Box 2 provides the essential reporting elements of each. If applied together, these should provide all interested parties with sufficient detail about the reproducibility and translatability of data, as well as the validity of the conclusions. This, in turn, has the potential to influence how research using animals is conducted globally, given that many researchers rely on publications to keep up to date with current research protocols, techniques and models. The HARRP incorporate and support the implementation of existing reporting standards as illustrated in Box 2 and build upon the ICLAS Ethical Guidelines for Researchers, Editors and Reviewers (http://iclas.org/committees/ethics-and-animal-welfare-committee).

There are now so many reporting guidelines that it is possible authors simply do not know which ones apply or when, and so they comply with none.

One of the criticisms of existing reporting standards is that they are written in technical language and written in English, which is not the primary language of many scientists. The HARRP, therefore, use non‐technical language to ensure that all parties involved understand which information must be clearly communicated in the Abstract, Introduction, Methods and Discussion sections. This aids transparency and could simplify translation of the principles into other languages—if any of the parties involved in the publication process does not fully understand the requirements of the reporting standards, it becomes difficult to make a meaningful assessment of whether papers are compliant. In areas of the world that lack regulation or a local framework governing the use of animals in research 6, the HARRP could be used as an easily translatable minimum standard, upon which a more structured framework could be built.

Another common criticism is that the exact detail of what should be reported varies between research disciplines and/or experimental design. The HARRP represent a first attempt to harmonize reporting across all research fields that depend on the use of animals, including preclinical research, fundamental biomedical research, toxicology and regulatory studies, wildlife studies and field work, agricultural research and veterinary studies. For this reason, the principles are not intended to replace or supersede the more detailed guidelines already in existence, but to weave these together to improve reporting in individual disciplines. By identifying the underlying concerns and utilizing common themes, the HARRP should provide all stakeholders (journals, databases, conferences, funders, researchers, research institutes, plus scientific and publishing bodies) with a reporting standard that is simple to implement.

## Adoption of HARRP

The experience from the CONSORT guidelines for clinical trial studies tells us that the unilateral adoption of a reporting standard by a significant number of journals can greatly help implementation by limiting the ability of authors to bypass such efforts through their choice of journal 7. ICLAS believes that the HARRP provide such a solution by defining the minimum reporting standards for animal‐based research. Given the evidence that efforts to implement reporting standards, such as the ARRIVE guidelines, are falling short of achieving their intent 3, the introduction of an aggregator minimum standard communicates to the research community and the public alike the benefits to improving animal research reporting in terms of reproducibility and reliability of animal studies. The availability of primary data allows the results to be included in secondary analyses, which maximizes the value from animals used in the research and potentially reduces their use in future experiments. It would also provide insurance against data loss, hardware/software malfunctions or out‐of‐contact authors. Thus, the HARRP communicate a non‐negotiable expectation that the standard of reporting for all animal‐based research must improve.

The number of papers reporting flaws in experimental design and limitations of animal models underline a need to better disseminate information about experimental protocols so that the whole research community can learn from it. Reporting of the study design is fundamental for ensuring valid and reproducible results. Reporting how the research was conducted depends on details of all procedures and materials to facilitate retrospective review of the protocol and, if necessary, inform changes to future studies. Details of experimental protocols can vary greatly but are all relevant for replication. Furthermore, housing, husbandry and other non‐experimental factors should be reported, because they are known to vary between laboratories and even within laboratories over time. As these factors may influence research results, they are equally important for interpreting individual or multiple studies.

Additionally, authors need to take reporting standards into account when planning and conducting their experiments; otherwise, it will require additional effort to provide the level of detail required. To address this point, the HARRP focus on a level of reporting consistent with legal minimum standards (where available) and generally accepted good practice for laboratory animal scientists. There should be nothing preventing authors working in countries that have established research frameworks from fulfilling and reporting these basic requirements.

## Setting global standards

Consistent with its aims to improve the quality of animal‐based research, ICLAS’ goal is to support all stakeholders, not just those in middle‐ to high‐income countries, to improve both the conduct and reporting of ethical animal‐based research. We are, therefore, keen to explore ways in which we can support the dissemination of the HARRP into countries where research is undertaken in the absence of specific regulatory, scientific or ethical guidelines or policies. Such an approach would provide a mechanism by which reporting standards could not only influence global standards of the treatment of laboratory animals, but also address the volume of poorly conducted or unethical research published in journals with substandard or absent review processes 8.

Scientists’ awareness of initiatives that aim to change cultural and behavioural aspects, such as those promoting data sharing as well as research reporting standards, is difficult to measure and may be complicated by other factors. There are now so many reporting guidelines that it is possible that authors simply do not know which ones apply or when, and so they comply with none. If authors do not recognize flaws with their own experimental design and analysis that reporting guidelines are designed to highlight, then the issues are compounded further. Thus, awareness needs to be tackled collectively through ongoing education and training to support researchers at all stages of their career and to educate them about pitfalls and how to avoid them in their own work 8.

Clinical field studies have shown that education in systematic reviews creates a quality awareness of all steps of the research process, inducing a motivation to improve performance of future research. At present, systematic reviews of animal studies are not easy to perform, because published reports often contain insufficient technical detail or are of insufficient quality to make the conduct of a meaningful review possible. Attention placed on good experimental design, encouraged through the need to report HARRP details, should mean that any future animal studies are properly reported so that data can be used in meta‐analyses. Any use of animals, such as protocol development, pilot studies, as a source of biological materials, or as “reagents” for *in vitro* studies should be reported. This is critical to ensuring that animals used are not wasted, that animal studies are not unnecessarily duplicated due to underreporting of research and that research can be repeated or verified if required 9.

These concepts were supported by delegates of the eighth World Congress on Alternatives and Animal Use in the Life Sciences held in 2011, who adopted the Montréal Declaration on the Synthesis of Evidence to Advance the Principles of the 3Rs in Science 10—calling for a change in planning, executing, reporting, reviewing and translating animal research. Funders can provide financial support to set up and maintain required resources, be that infrastructure, systems or education and training. They can also, as many already do, set out clear expectations regarding good practice as a condition of funding and enable the fulfilment of such expectations. Research institutions, journals, scientific and publishing societies can also ensure clear expectations regarding research reporting standards, as well as provide training opportunities. They can also work with other stakeholders to help disseminate, raise awareness of and reward good research practice.

So far, a clear strategy for identifying and addressing the real or perceived barriers within scientific cultures that continue to hinder progress in improving the use of animals in research and commensurate reporting standards has been missing. ICLAS recognizes that there is no “ideal”, one‐size‐fits‐all solution and stakeholders around the globe will need to tackle their own unique combination of issues relating to the culture and practice of scientific research. An immediate and unilateral worldwide commitment by all stakeholders to enforce the HARRP could provide the momentum to improve the practice and reporting of animal research and ultimately fulfil contemporary best practice reporting standards such as NIHPGRPR and ARRIVE.
